# Digital Filtering Techniques Using Fuzzy-Rules Based Logic Control

**DOI:** 10.3390/jimaging9100208

**Published:** 2023-09-30

**Authors:** Xiao-Xia Yin, Sillas Hadjiloucas

**Affiliations:** 1 Cyberspace Institute of Advanced Technology, Guangzhou University, Guangzhou 510006, China; xiaoxia.yin@gzhu.edu.cn; 2 Division of Bioengineering, School of Biological Sciences, University of Reading, Reading RG6 6AY, UK

**Keywords:** fuzzy filter, image processing, color image sequences, multichannel filtering, neuro-fuzzy network

## Abstract

This paper discusses current formulations based on fuzzy-logic control concepts as applied to the removal of impulsive noise from digital images. We also discuss the various principles related to fuzzy-ruled based logic control techniques, aiming at preserving edges and digital image details efficiently. Detailed descriptions of a number of formulations for recently developed fuzzy-rule logic controlled filters are provided, highlighting the merit of each filter. Fuzzy-rule based filtering algorithms may be designed assuming the tailoring of specific functional sub-modules: (a) logical controlled variable selection, (b) the consideration of different methods for the generation of fuzzy rules and membership functions, (c) the integration of the logical rules for detecting and filtering impulse noise from digital images. More specifically, we discuss impulse noise models and window-based filtering using fuzzy inference based on vector directional filters as associated with the filtering of RGB color images and then explain how fuzzy vector fields can be generated using standard operations on fuzzy sets taking into consideration fixed or random valued impulse noise and fuzzy vector partitioning. We also discuss how fuzzy cellular automata may be used for noise removal by adopting a Moore neighbourhood architecture. We also explain the potential merits of adopting a fuzzy rule based deep learning ensemble classifier which is composed of a convolutional neural network (CNN), a recurrent neural networks (RNN), a long short term memory neural network (LSTM) and a gated recurrent unit (GRU) approaches, all within a fuzzy min-max (FMM) ensemble. Fuzzy non-local mean filter approaches are also considered. A comparison of various performance metrics for conventional and fuzzy logic based filters as well as deep learning filters is provided. The algorhitms discussed have the following advantageous properties: high quality of edge preservation, high quality of spatial noise suppression capability especially for complex images, sound properties of noise removal (in cases when both mixed additive and impulse noise are present), and very fast computational implementation.

## 1. Introduction

The quality of color image data is often degraded by additive noise, impulse noise, or mixed additive and impulse noise [1]. Additive noise, as a temporal type of noise source, includes dark noise, shot noise, and noise from mechanical vibrations, which, generally, can be approximated by a Gaussian function with a signal-dependent mean [2]. Impulse noise is normally produced by noisy sensors and/or transmission errors [3,4,5,6] in the data transmission or communication channels, as well as during the data capturing process from digital cameras [7]. Impulsive noise can be characterized by short duration, high-energy spikes, with independent random process statistics, and more accurately modelled by heavy-tailed, non-Gaussian distributions [8,9]. Although there are many impulse noise removal techniques [10,11,12], as more recently discussed by Roy et al., it is possible to efficiently remove impulse noise from grey-scale images using a support vector machine (SVM) classifier in conjunction with a fuzzy filtering technique [13]. Furthermore, a neuro-fuzzy network may be used for de-noising [14] or hybrid approaches [15,16]. A short overview of different denoising methods applicable to image processing and image understanding can be found in the work by Tian et al. [17] and the work by Fan et al. [18].

The issue in properly fixing the challenges of color image denoising is to (a) pinpoint the origins of the noise characteristics and account for their diversity, and (b) take into account the non-stationary statistics of the underlying image structures [19]. These two factors have sparked a recent interest into combining the noise and/or structure estimation with color image restoration filtering algorithms. When constructing filters for color image restoration, three key goals must be satisfied: noise reduction, chromaticity maintenance, and edge detail preservation [20].

It is well established that problems related to signal processing cannot be efficiently dealt with by applying linear techniques, especially when it comes to the nonlinearity of image formation processing. Furthermore, one has to cover the nonlinear nature of the human visual system [21]. By combining standard linear and nonlinear filters with fuzzy logic, fuzzy modelling serves as a link between linear and nonlinear approaches. Because information gathered from data can be contaminated by noise, establishing a correct mathematical model of a nonlinear system is more challenging, as it includes evaluating a deterministic component, a distinct stochastic component, and an increasing of parameters. In this case, fuzzy reasoning is highly suitable since it allows for the selection of soft thresholds that can better adjust to the nonlinearity in the model inputs [21,22].

Fuzzy inference refers to the formulation process of the mapping from a given input to an output via fuzzy logic statements. Then, the mapping establishes a foundation where decision making can be realized, or patterns are to be identified [23]. The fuzzy rule-based modelling approach is grounded in verbally formulated rules, which are overlapped in the whole parameter space [24,25]. The goal of the fuzzy modeling approach is to create a fuzzy rule foundation that is suitable for the task at hand. The filtered output is flexible to adjust, and nonuniform patterns which are most similar to edge and corner in local structure are treated. A window-based fuzzy filter is typically used to recover and rectify a corrupted pixel locally, where the fuzzy rule responds immediately with the signal elements within the operational window.

In summary, a logic control strategy can be implemented as part of computational intelligence methods to process and analyse highly nonlinear, time-varying images and signals in a natural and uniform way with the use of fuzzy set theory. The purpose of this paper is to clarify how to incorporate fuzzy rule based on traditional vector and fuzzy vector spaces to algorithms tailored for digital image filtering. All the main definitions and properties of fuzzy vector spaces available in the literature are systematically covered and fundamental differences between vector and fuzzy vector spaces are highlighted. The paper also makes a contribution to current color image analysis techniques, by presenting a survey on comparative performance evaluations of methods proposed in the literature, discussing the applicability of different fuzzy methods to improve the effectiveness and the quality of data analysis, from the perspective of image denoising, image restoration, image classification, edge detection, and other well established performance metrics. In addition, the paper also provides a step by step approach for the analysis and synthesis of fuzzy-model-based nonlinear filters and provides new directions for the development of ensemble model filters based on several base learning techniques such as convolutional neural network (CNN), recurrent neural networks (RNN), long short term memory neural network (LSTM) and gated recurrent unit (GRU) approaches, all within a fuzzy min-max (FMM) ensemble.

This article is suitable for researchers, practitioners, engineers and educators in the field of artificial intelligence in general, with prior familiarity with specific topics such as image/signal processing or categorization. We also assume readers have a basic understanding of classical logic concepts like truth tables and logic operations, but not necessarily about their many-valued or fuzzy logic counterparts which are reviewed in this article. The main goal is the clarification of expert-system-like rule sets based on fuzzy sets in order to support the development of new algorithms. This framework is, therefore, conceived to be a potential starting point to a future standard framework for guiding experts in adopting fuzzy rule based inference engines to enhance the use of computational intelligence techniques for multi-channel color image analysis and classification.

The following provides an overview of how this paper is structured. The main framework for color image analysis is presented in Section 2. In this section, the noise models used for the evaluation of the fuzzy filter performance are presented. Section 3 overviews fuzzy rule design on the basis of local information. Different types of fuzzy linguistic variables are also considered. Section 4 addresses the construction of a fuzzy filter. Experimental analysis and comparisons, in terms of quality performance are provided in Section 5. Finally, Section 6 summarizes the most significant aspects highlighted in each section and provides concluding remarks and directions for future research.

## 2. General Filtering Framework for a Color Image

It is well acknowledged that color imaging transmits more information than greyscale imaging. Consequently, most scientific applications adopt color and multi-spectral imaging equipment. The origins of this obvious benefits can be linked back to Fellgett’s multiplex advantage in astronomy, which indicates that a multispectral system has an added benefits over its monochromatic counterpart due to the higher throughput per unit time related to the various individual channels of the collected data. Noise filtering is one of the most typical image processing operations in the framework of integrating data, and it is an important aspect of any image processing system [26,27].

### 2.1. Impulse Noise Models

Digital color images are modelled in a specific color space, that is, RGB, L${}^{*}$a${}^{*}$b${}^{*}$ or HSV. The RGB color space is employed in a large percentage of applications. Various colors can be obtained via blending red (R), green(G) and blue (B) light in diverse percentages [28]. Assuming [x] denotes a chromatic R, G, B image, each pixel $x_{k}=\left(x_{k}^{R},x_{k}^{G},x_{k}^{B}\right)$ then denotes a three-component vector in a color space. Moreover, an address *k* denotes a location in [x], which is referred to as a pixel or picture element. It is noted that in a color image, each individual channel *L* can also be totally regarded as a monochrome (grayscale) digital image $[x^{L}],L=R,G,B$. The correlation existing among the colour components of natural images [29] is represented by the color attributed in the individual pixels in an image. The (gray scale) intensity is typically stored as an 8-bit integer. It generally gives 256 probable diverse grey shades, from black to white, usually reflected as a [0,255] integer interval.

For the evaluation of fuzzy noise suppression algorithms, there are three commonly used models to simulate different types of distortions for a multichannel signal corrupted by impulsive noise [7,27,28,30].

Let $x_{k}=\left[x_{k}^{R},x_{k}^{G},x_{k}^{B}\right]$ model the noisy pixel, where

(1) $$x_{k}^{L}=\left\{\begin{array}{c} \upsilon_{k}^{L}\;\;\mathrm{with}\;\mathrm{Probability}\;\pi \\ \iota_{k}^{L}\;\;\mathrm{with}\;\mathrm{Probability}\;1-\pi \end{array}\right.$$

and the contamination component $\iota_{k}^{L}$ is a stochastic noise term [7,27,28,30].

The following noise models are assumed:

(I) Salt and pepper noise (fixed-valued noise):

In this kind of noise model, pixels in the image vary considerably in color compared with their surrounding pixels. The color of a noisy pixel is rarely related to the color of its surrounding picture elements.

(a) Let $\left\{\upsilon_{k}^{L}\right\}$ with L=R,G,B model the original pixel before being vitiated by the noise processing. And the picture elements are subsequently distorted as per the scheme below:
  (2) $$x_{k}=\left\{\begin{array}{c} \{\upsilon_{k}^{L}\}\;\;\mathrm{with}\;\mathrm{Probability}\;1-p\\ \{\iota_{1},\upsilon_{k}^{G},\upsilon_{k}^{B}\}\;\;\mathrm{with}\;\mathrm{Probability}\;p_{1}p\\ \{\upsilon_{k}^{R},\iota_{2},\upsilon_{k}^{B}\}\;\;\mathrm{with}\;\mathrm{Probability}\;p_{2}p\\ \{\upsilon_{k}^{R},\upsilon_{k}^{G},\iota_{3}\}\;\;\mathrm{with}\;\mathrm{Probability}\;p_{3}p\\ \{\iota_{1},\iota_{2},\iota_{3}\}\;\;\mathrm{with}\;\mathrm{Probability}\;p_{4}p \end{array}\right.$$
  where $\iota_{1},\iota_{2},\iota_{3}$ are independent and equal to 0 or 255, *p* is the sample corruption probability with $p=1-{\left(1-\pi \right)}^{3}$ and $p_{1},\;p_{2},\;p_{3}$ are corruption possibilities regarding every color channel, so that $\sum_{i=1}^{4}p_{i}=1$. In such circumstances, the contamination of color image components is still non-correlated [7,27,28,30].
(b) In this kind of noise model, the RGB channels are corrupted due to the impulse noise (0 or 255) and possibility *p* like in (a), while the contamination process is correlated. This case is called the *extended noise model* and has been discussed extensively in [27,30,31,32]. Its main characteristic is that $p_{i}=0.25$, i=1,2,3,4. This implies that one of the image channels will probably be corrupted since an additional channel has been corrupted already.

(II) Impulsive uniform or randomly valued noise: In such a case, the average value is equal to the real one. Let $x_{k}=\left\{\iota_{1},\iota_{2},\iota_{3}\right\}$ with probability *p*, where the value of a noisy pixel from each color channel $\iota_{1},\iota_{2},\iota_{3}\in \left[0,255\right]$ is modelled as a random process, being identically distributed and independent, with an arbitrary potential probability density function. The contamination of the color image components is uncorrelated.

### 2.2. Window-Based Filtering for Fuzzy Inference System

Natural images are modelled as non-stationary processes. Filtering is normally performed in a series of stationary sub-images, through the subdivision of an image into smaller regions [33,34]. These smaller image regions are identified via a support window W. Over the image domain, the filter window W is moved to individually modulate all those image pixels, aiming to process the input image thoroughly. It is also referred in the literature as a sliding windowing procedure [34].

A fuzzy filter operation is usually based on windows. For every picture element of the noise image, a series of adjacent picture elements is taken into consideration. Fuzzy operators process these neighborhood data via fuzzy rules to speculate calibration terms aimed at removing noise. If none of the rules are met, the center pixel remains largely unchanged [35]. More specifically, according to [35], x(c) denotes the image element luminance at the center location *c* in the noise image and let $W\left(c\right)=x_{j}\left(c\right);j=1,\ldots ,\left(2D+1\right)\times \left(2S+1\right)$ be the set of neighboring pixels which belong to a (2D+1)×(2S+1) window around x(c). With reference to [35], the input variables of the operator are the luminance differences defined by

(3) $$\nabla y_{k}\left(c\right)=y_{k}\left(c\right)-x_{k}\left(c\right)$$

The resulting luminance value $y_{k}=x_{k}+\nabla y_{k}$ is generated by the combination of the output variable ∇y(c) which is the correction term, and x(c). Here, *k* labels the pixel position in an image. To achieve the goal of yielding the correction term with removal of the noise impulses, the operator nonlinearly maps the set of input parameters to the output parameter by using fuzzy rules. Given that images to be processed have *l* grey values, then input and parameters take values in the interval [−l+1,l−1]. Fuzzy set theory allows for the stepwise evaluation of the element membership in a set after applying a subordinate function valued at the real unit interval [0,1]. Fuzzy rules cope well with a significant range of patterns in the pixels when detecting noise impulses [36].

## 3. Basic Concepts of Vector Filters

Noise reduction in multi-channel images has been extensively studied over the past few years, mainly because of its significance to chromatic image processing [27,37]. The certain fundamental concepts and defined notations with regard to chromatic images provide the basis for development and evaluation of fuzzy-rules-based filtering techniques.

Multi-channel images become vector-valued samples rather than scalars. However, vector-valued samples cannot be sorted directly for their vector structures, leading to difficulty in noise removal from an image. Barnett [38] has proposed four vector-valued sub-ordering schemes: the first is marginal ordering (M-ordering), the second is reduced (aggregated) ordering (R-ordering), the third is partial ordering (P-ordering) and the fourth is conditional ordering (C-ordering). R-ordering schemes are frequently used to acquire order statistics by mapping an observed vector-valued sample to a scalar quantity, which can be viewed as the aggregated distance between one sample and the others.

### 3.1. Ranking Vector-Valued Data

Distinct distance measure metrics (DDMM) may be adopted to develop higher order statistics for multichannel filtering techniques [9,34,39]. We can assume a situation where a filtering window (or a sliding window) with size $N={\left(2\tau +1\right)}^{2}$ (*N* is generally odd) is applied on the image I centered at location (c). The generalized Minkowski metric is the most frequently employed method to realize the quantification of the distance between two three-channel samples $x_{i,L}$ and $x_{\left(j,L\right)}$ with L=R,G,B is the generalised Minkowski metric, written as

(4) $$\rho \left(x_{i},x_{j}\right)=\left|\right|x_{i}-x_{j}{\left|\right|}_{\gamma }={\left(\sum_{L=R,G,B}{\left|x_{\left(i,L\right)}-x_{\left(j,L\right)}\right|}^{\gamma }\right)}^{\frac{1}{\gamma }}$$

where γ denotes the norm parameter, with γ=1,2 and *∞* denoting $L_{1}$ norm of Block distance, $L_{2}$ norm of Euclidean distance, and $L_{\infty }$ norm of Max distance, respectively. The norm $L_{2}$ for the Euclidean distance measures is frequently used. In this context, the expression $\left|\right|\cdot {\left|\right|}_{L_{2}}$ is rewritten as ||·|| with omitted $L_{2}$ for convenience [9,34,39].

The vector angular distance between two three-dimensional vectors, $x_{\left(i,L\right)}$ and $x_{\left(j,L\right)}$, in the orientation processing of chromatic pictures, in which L=R,G,B is defined as the Equation (5) [9,34,40]

$$\begin{array}{c} \rho \left(x_{i},x_{j}\right)=\cos^{-1}\left(\frac{x_{i}\cdot x_{j}}{\left|\right|x_{i}\left|||\right|x_{j}\left|\right|}\right)=\cos^{-1}\left(\frac{\sum_{L}x_{\left(i,L\right)}x_{\left(j,L\right)}}{\sqrt{\sum_{L}x_{\left(i,L\right)}^{2}}\sqrt{\sum_{L}x_{\left(j,L\right)}^{2}}}\right). \end{array}$$

where L=R,G,B. The R-ordering of input vector $x_{1},x_{2},\ldots ,x_{N}$ is determined from the resulting order statistic, which is the aggregated distance corresponding to $x_{i}$

(5) $$\begin{array}{c} d_{i}=\sum_{j=1}^{N}\rho \left(x_{i},x_{j}\right). \end{array}$$

The resultant R-ordering is a ranked vector $x_{\left(1\right)}\leq x_{\left(2\right)}\leq \ldots \leq x_{\left(N\right)}$, with the identical order as sorted $d_{\left(k\right)}$, k=1,2,…,N where

(6) $$\begin{array}{c} d_{\left(1\right)}\leq d_{\left(2\right)}\leq \ldots \leq d_{\left(N\right)}. \end{array}$$

The nonlinear ordered multi-channel estimator defines the vector $x_{\left(1\right)}$ as the filter output [41].

### 3.2. Vector Directional Filters

Basic vector directional filter (BVDF) [40] and generalised vector directional filter (GVDF) [40] are two extensively adopted families of directional filters. They form the basic methodological foundation, which enable image processing techniques to improve the capability in the detection and classification of objects and feature extraction from an image [42]. The aggregated angular distance between the input vector and its neighbors is minimized by the output of BVDF, which also removes image vectors with non-typical orientations in the vector space. The BVDF output $y_{BVD}$ is the lowest ranked vector $x_{\left(1\right)}$. The GVDF is designed in two stages. The first stage aims to match the first *r* terms of ranked input vectors according to the aggregated angular distance. The second stage adopts an additional filtering procedure processing this set of color vectors $x_{\left(i\right)}$, i=1,…,r according to their magnitude.

Further methodologies that are used for color vector directional calculation are presented by Bhatti et al. [42]. In their research work, a satellite image dataset that consists of different color intensity with a large amount of noise is analysed. The satellite image dataset is viewed as the complex numbers, and represented as a quaternion fourtuple (w, x, y, z). Under the RGB color model, each pixel of a color image can be represented as a pure quaternion, with an extra dimension of time. Quaternion Fourier Transform and Clifford algebra are the two main algorithms to be used in color vector directional filtering.

#### 3.2.1. Directional-Distance Filters

A directional-distance filter (DDF) was put forward in [43] for improving the directional filter efficiency. Such a filter preserves the BVDF structure and uses a diverse distance criterion to sort the vectors within the process window [43]:

$$\begin{array}{c} d_{\mathrm{DDF}}={\left(\sum_{j=1}^{N}\left|\right|x_{i}-x_{j}\left|\right|\right)}^{1-p}\cdot {\left(\sum_{j=1}^{N}\cos^{-1}\left(\frac{x_{i}\cdot x_{j}}{\left|\right|x_{i}\left|||\right|x_{j}\left|\right|}\right)\right)}^{p} \end{array}$$

where i=1,2,…,N.

The advantage of the directional-distance filter is the capability to remove the noise and repair the images without introducing a significant distortion. The drawback is that these direction based filters present a high computational complexity [44]. In the research conducted by Atitallah et al. [44], an optimized field-programmable gate array (FPGA) (hardware) was designed in combination with the adaptive vector directional distance filter (AVDDF) for removing noise from the images in real-time.

#### 3.2.2. Hybrid Multichannel Filters

Hybrid multichannel filter (HMF),which is another efficient rank-ordered technique, was discussed in [45]. In [46], an alternative approach to HMF is mainly discussed. Simultaneously, the vector magnitude and orientation can be incorporated within hybrid multichannel filters. This way, further performance improvement of traditional filters may be achieved. The output of an HMF ($y_{\mathrm{HM}}$) consists of the outputs of a VMF ($y_{\mathrm{VM}}$) and a BVDF ($y_{\mathrm{BVD}}$) filters [45]. It is formulated as follows

(7) $$\begin{array}{c} y_{HM}=\left(1-\alpha \right)\beta y_{BVD}+\left(1-\beta \right)y_{VM}+\alpha \beta y_{\xi } \end{array}$$

where α, β∈[0,1]. $y_{\xi }$ is the center vector-valued picture element of a filtering window. $y_{\mathrm{BVD}}$, $y_{\mathrm{VM}}$, and $y_{\xi }$ denote the outputs of a BVDF, a VMF, and an identity filter, separately [37].

One more hybrid basic vector filter and its switching extensions are introduced by Zhong et al. [47], as an extension of the above. By utilizing reliable marginal filter and retaining the inherent correlation between multi-channels, the proposed method selects the vector, with minimal distance to the output of the marginal median filter. Based on this scheme, some well-known switching filters are easily modified to improve their noise suppression capability.

#### 3.2.3. Center-Weighted Vector Median Filters (CWVMFs)

While center-weighted vector median filters (CWVMFs) [31,45,48] relate to the simplest case filters which are only controlled by the value of the center weight, CWVMFs use a positive integer central weight, *w*, the output of which is written as

(8) $$\begin{array}{c} y_{w}\left(c\right)\in W\left(c\right)|=\arg\min_{1<i<N}\left\{R_{i}^{w}\right\} \end{array}$$

where W(*c*) denotes a sliding window with a size *N* centered at position *c*, and the sign $R_{i}^{w}$ labels the central weighted aggregated vector distance (CWAVD), according to [49].

Even though the standard VMF is powerful enough for noise suppression, some details are to be smeared in the filter window. With an increase in the value of *w*, the detailed retention ability of the filter is uplifted whereas the noise inhibition property is reduced. When w≥(N+1)/2 [50], the CWVM filter will definitely become an identity filter.

More recently, a better version named center-weighted trimmed vector median (CWTVM) filter was proposed [50]. A CWTVM filter allows more robust local structure classification than the CWVM filter when high central weights are involved. The CWTVM filter ranks in a specific way, which is to rank the picture elements of a filtering window in the ascending order. And this ranking is based on their Euclidean distances from the CWTVM filter to the center pixel of the window (center local distance ranking order). Pixels are arranged within a particular center pixel and local distance order is utilized as inputs for the filter output to be calculated.

As the central weight is increased, the proposed CWTVM filter can obtain much more stability in filtering impulsive noise in contrast to the CWVMF, which receives a benefit from its center-distance trimmed technique.

As the CWVM filter’s alternative version, the switch-based CWVMF is also worth discussing. Suppose that there is a filter window W(c) centered at the picture element x(c) that contains *N* image element specimens, those filters initially adopt values of CWVMFs with the central weights w=1,2,…,(N−1)/2, separately, to gain a set of reference estimates, $\left\{y_{w}\left(c\right)|w=1,2,\ldots ,\left(N-1\right)/2\right\}$, from the picture elements of the window, in which $y_{w}\left(c\right)$ means the output of a CWVMF with a central weight *w*. Afterwards, the distance from the center pixel to every referential estimate is identified.

A drawback of this filter is that the accuracy of their binary noise detection heavily relies on the threshold values and the condition (e.g., impulse noise model and image structure) of the threshold optimization. Furthermore, the switch-based reconstruction mechanism works only efficiently in the removal of the impulse noise. It has to be noted that such practice is not suitable for suppressing other kinds of noise, suc as additive noise or mixed noise that may also contaminate images as.

#### 3.2.4. Peer Group Techniques

A peer group filter (PRT) is put forward in [51], and more applications are discussed in [52,53]. The main objective of the peer group filter is to suppress impulse noise while preserving important image features like edges, corners and textural information. The peer group P associated with the central pixel of W is a set consisting of the central pixel x(c) and its neighbour pixels belonging to W, whose distance to x(*c*) is not exceeding *d*. The parameter *r* denotes the number of neighbours of x(*c*), which belong to the peer group P(x(c),r,d).

A fast peer group filter (FPGF), introduced in [54], just works like a simple switching filter performing between an identification filter and VMF. The center pixel x(c) is retained when there are *r* neighbors in W, based on the distance parameter *d*. Or else, the vector medium $x_{\left(1\right)}$ of the samples in W will substitute for the center pixel. After that, the filter output is $y_{\mathrm{PG}}\left(c\right)$. And it is then formulated according to [54] as follows

(9) $$\begin{array}{c} y_{\mathrm{PG}}\left(c\right)=\left\{\begin{array}{c} x(c)\;\;\mathrm{if}\;\mathrm{all}\;x(c)\in P(x(c),r,d)\\ x_{\left(1\right)}\left(c\right)\;\;\mathrm{otherwise} \end{array}\right. \end{array}$$

Simplicity and accelerated computational performance are the main merits of the proposed FPGF approach. In addition, according to the similarity to the center pixel [51,55,56], ordered neighbor pixels are used to define the peer group. Color pixel vectors in a filter window $x_{i}$, $i\in 0,\ldots ,n^{2}-1$ are ordered to $x_{\left(i\right)}$, $i\in 1,\ldots ,n^{2}-1$. Let $x_{0}$ instead of x(c) denote the center pixel vector (as explained in [51,55]) and the similarity measurement μ can be expressed using the following inequality:

(10) $$\begin{array}{c} \left\{\begin{array}{c} \mu \left(x_{0},x_{\left(0\right)}\right)\geq \mu \left(x_{0},x_{\left(1\right)}\right)\geq \ldots \geq \mu \left(x_{0},x_{\left(n^{2}-1\right)}\right) \end{array}\right. \end{array}$$

It is obvious that $x_{0}=x_{\left(0\right)}$, and

(11) $$\begin{array}{c} P\left(x_{0},r,d\right)=\left\{x_{\left(i\right)},i=1,\ldots ,r\right\} \end{array}$$

The number of members of *r* may be further calculated by using Fisher’s linear discriminant (FLD). Based on the discussion above, two important parameters determine the appropriate construction of a peer group filter: distant threshold *d* and the number of peer group members *r* [51,55].

Generally, there are several vector filters in color image processing which take advantage of correlations existing across different color image channels. Furthermore, many of these non-linear vector filters are taking advantage from the recent developments in the theory of robust statistics [57,58]. Considering the use of well ordering principle, the lowest ranked vectors are viewed as the filter output of these filter structures. This ranked order is derived from the results acquired following the use of the distance or similarity measure criteria adopted [30,34], where the lowest ranked vectors are closest to all the other vectors in the filter window. Alternatively, a reduced order of vector means the importance of the central vector is increased, so that its likelihood to be the lowest ranked vector (filter out) is the highest.

It is feasible to customize the smoothing criterion to local image characteristics rather than using typical vector filter for a predetermined amount of smoothing or noise removal [34]. There have been several approaches discussed in the literature that achieve this through (i) vector combination in a sliding window [34,59]; (ii) weighted vector median operations [60,61,62]; (iii) cluster analysis when a noisy vector detection is required [63,64,65,66]; (iv) combination of different vector filters in each image location by using a rational function for the final output [67]; (v) Other signal processing methods such as wavelet transforms and curvelet transforms [68,69,70,71] together with neuro-fuzzy filters [72,73,74] are also possible.

## 4. Fuzzy Vector Filters

To create a fuzzy filter, it is crucial to understand where and how a special membership function is formed, how it is used, and how its influence is measured, as well as how it can be tailored according to the imaging situation at hand to produce meaningful results. Solutions to the issue of membership definition and graded membership [9] may be various, according to distinct interpretations of fuzziness. The following subsections first review a few fundamentals and define fuzzy (logic) variables regarding color images. The approaches adopted to calculating specific fuzzy variables are represented as special cases tailored to specific problems. These lead to relevant membership functions suitable to achieve outputs of the fuzzy logic filters.

### 4.1. Standard Operations of Fuzzy Set

On X, the fuzzy set is defined, and based on the color value in an image [75], the imprecision is indicated by the subordinate function μ. $\mu_{X}\left(x\right)$ is the subordinate function that gives a precise definition regarding how the values of *x* in X will be mapped within [0,1].

**Figure 1:**
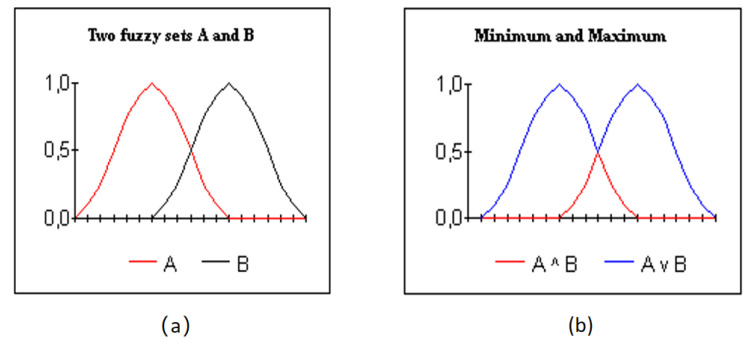
Illustration of T-Norm (**a**) and S-Norm (**b**).

Similarly to classical set theory, fuzzy sets apply functions *T*-norm and *S*-norm (or *T*-conorm) to enable intersection and union operators. A *T*-norm, called ltriangular norm, is an operator *T* from [0,1]×[0,1] into [0,1] which is transitive, associative and elevating in both variables while admitting 1 as the unit element. It stands for a conjunction and at the same time generalizes the logical proposition ‘and’, labeled by the intersection. This can be illustrated in Figure 1. The minimum operator of two fuzzy sets A and B, shown in Figure 1a is the model intersection. It is the red line as shown in Figure 1b. This minimum operator is one of the “triangular norms”. With the T-norms there are the T-conorms, also called the S-norms. They model union. The maximum operator is an S-norm. It is the blue line as shown in Figure 1b.

A *S*-norm, is an operator *S* from [0,1]×[0,1] into [0,1], which is referred to as triangular conorm. It is commutative, associative and elevating in both variable quantities which have the attached property that allows 0 as the identity element. It theoretically denotes a disjunction and generalises union and also the logical operator ‘or’ at the same time, labeled by union.

According to [76], the minimum and the maximum functions labelled by min(A,B) and max(A,B) separately, are also used as models to define intersection and lunion of fuzzy sets (A,B∈[0,1]). The sign ‘min’ corresponds to the greatest *T*-norm and ‘max’ corresponds to the least *S*-norm or *T*-conorm. Additionally, according to [77], the product and the probabilistic sum are also another way to express *T*-norm and its dual *T*-conorm, i.e.,

(12) $$\begin{array}{ccc} & & \Pi (A,B):=AB\;\;A,B\in [0,1] \end{array}$$

(13) $$\begin{array}{ccc} & & \Pi^{\prime }\left(A,B\right):=A+B-AB\;\;A,B\in [0,1]. \end{array}$$

A complementation (or a standard negation) is an operator *c* from [0,1] into [0,1] which is stringently decreasing, such that c(0)=1, c(1)=0. The most-utilized complementation [78,79,80] is:

(14) $$\mu_{c(A)}\left(x\right)=1-\mu_{A}\left(x\right)\;\;\mathrm{for}\;\forall x\in [0,1].$$

The different *T*-norm, *S*-norm and complementation operators make it possible to map from inputs to outputs while utilizing a series of linguistical rules. These rules hold the form IF *A* AND *B* OR *C* THEN. The *T*-norm and *S*-norm operators merely replace the AND and OR operators, accordingly. The merit of such inference system lies in that it is quite intuitive, largely reducing the necessary time for optimizing the output function [81].

### 4.2. Fuzzy Approaches for Noise Identification

A principal merit of order statistics filtering is its ability to incorporate noise-identification algorithms with sophisticated filtering methods [81,82]. Order statistics filtering embodies the main ideas behind vector median filters. An alternative method for impulsive noise identification of chromatic images is proposed in [83]. Standing on the opposite side of the vector methods, this fuzzy noise identification is utilized in every chromatic component respectively. With application of n×n filtering window, for every chromatic component of every image element, the absolute value diversities between the center image element $x_{0}$ and every chromatic neighboring pixel $x_{k}$. A fuzzy set small labelled by $S_{1}$ is designed to identify when these differences can be considered as sufficiently small (below a set of threshold value). $S_{1}\left(\Delta x_{k}^{R}\right)$ for $k=1,\ldots ,n^{2}-1$ are sorted in decreasing order and the *K* neighbors are taken into consideration only when they show sufficient differences.

In a similar manner, a membership function $S_{2}$ can be designed to investigate the correlation between individual color components. The local differences among three color components can be viewed as different fuzzy varieties which can therefore be evaluated by the fuzzy membership function [83].

(15) $$\begin{array}{c} \mu_{k}^{RG}=S_{2}\left(|S_{1}\left(\Delta F_{k}^{R}\right)-S_{1}\left(\Delta F_{k}^{G}\right)|\right)\\ \mu_{k}^{RB}=S_{2}\left(|S_{1}\left(\Delta F_{k}^{R}\right)-S_{1}\left(\Delta F_{k}^{B}\right)|\right) \end{array}$$

where the functions $\mu_{k}^{RG}$ and $\mu_{k}^{RB}$ are used to label the degree where the local difference in the R component resembles the local difference in the G and B components (between the central pixel and the image element at location *k*). The $\mu_{k}^{RG}$ and $\mu_{k}^{RB}$ are sorted in a descending order for the sake of assessing the degree of joint similarity in contrast to *K* neighbors and to avoid a probably noisy component being taken as reference when assessing the degree of similarity between local pixels and their differences [83].

Therefore, a chromatic component is taken as noise-free when

(a) it resembles certain neighbour values
(b) the local differences compared with neighbours resemble the local differences in certain other chromatic components
(c) the resemblance levels of the other RGB component values compared with the neighbour values are high. It is crucial not to introduce a probably noisy component as reference when calculating the similarity between the local differences.

#### 4.2.1. Fixed Valued Impulse Noise

In [7,28,84] histogram-based approaches to remove impulse noise have been discussed for fixed-valued impulse noise reduction. According to [84], (i) if an image is contaminated by a fixed-valued noise type, the noise histogram only contains peaks; (ii) if an image is contaminated by a mix of fixed-valued impulsive noise (FVIN) and Gaussian noise, and if the relative histogram involves both peaks and some other features around some extreme values, one can assume that a pixel can be identified; (iii) otherwise, it can be viewed as an image without a fixed value of impulse noise pixel.

The estimated standard deviation can be viewed as a fuzzy variable to identify individual the “impulse noise pixels” (introduced in [85]). In most cases, the low standard deviation corresponds to histograms which contain purely peaks while for the large standard deviation, it corresponds to histograms which contain peaks with certain features normally.

Plots of intensity value (horizontal-axis) with the largest quantity of detection (perpendicular-axis) in the histogram are used to decide if there are any peaks. A threshold value is then defined for tracing and identifying a proper peak. This value has been experimentally derived as 0.08 in [7].

According to [28], histograms come from the color components that are most likely corrupted with impulse noise. The reason lies in that pixels corrupted with fixed value of impulse noise are vastly different from surrounding pixels in a local window, leading to the minimum and maximum intensity values, frequently denoted by $q_{\max}$ or $q_{\min}$. To compute histograms for each image component, a two-step procedure is used relying on this concept: (i) the separation of the broken greyscale images into small chunks from each color channel, with a recommended chunk size of 5×5; and (ii) the determination of the value of the two featured intensities $q_{\max}$ or $q_{\min}$, which should be included in the calculation of the histogram, depends on the following conditions to be satisfied according to [28]: if $q_{\max}$ is involved, then $|m_{1}-q_{\max}\left|\;>\;\right|m_{1}-m_{2}|$ or $|m_{2}-q_{\max}\left|\;>\;\right|m_{1}-m_{2}|$; if $q_{\min}$ is involved, then $|m_{1}-q_{\min}\left|\;>\;\right|m_{1}-m_{2}|$ or $|m_{2}-q_{\min}\left|\;>\;\right|m_{1}-m_{2}|$, where $m_{1}$ and $m_{2}$ denote the image intensities at two different locations within a local window with $m_{1}>m_{2}$.

Defining the subordinative degrees $\mu^{noise}\left(p_{R}\right)$ for the intensity value $p_{R}^{k}$ of a R component within the fuzzy set *noise* (or *large* denoted by ${\left[\cdot \right]}^{L}$ or $\mu^{L}$) [28]:

(16) $$\begin{array}{c} \mu^{noise}\left(p_{R}^{k}\right)={\left[\frac{H(p_{R}^{k})}{\sum_{p_{R}=0}^{255}H\left(p_{R}\right)}\right]}^{L}=\mu_{L}\left[\frac{H(p_{R})}{\sum_{p_{R}=0}^{255}H\left(p_{R}\right)}\right] \end{array}$$

For this constant value of impulse noise, it is expected that at least one intensity value with a subordinative degree in the fuzzy set *noise* can be identified successfully, or a conclusion can be made that image is not corrupted with the definite value of impulsive noise. If this is not the actual case, then the arbitrary value of impulse noise has to be detected.

#### 4.2.2. Random Valued Impulse Noise

The histogram-based approach discussed in [28] illustrates the fuzzy derivative with the histogram that is conducted in order to achieve pixel screening for RVIN. The fuzzy derivative is employed to evaluate the parameters of membership function. Here, we discuss the extension of this algorithm using a fuzzy variable that focuses on the evaluation of the differences existing between the chromatic components in special surroundings around a filtered central picture element. This approach aims to utilize such information to filter the color components of the central pixel, while preserving the observed diversities.

Owing to the different intensity values in distinct components [28]

(17) $$\begin{array}{ccc} RG(k_{1},k_{2}) & = & C_{R}\left(k_{1},k_{2}\right)-C_{G}\left(k_{1},k_{2}\right)\\ RB(k_{1},k_{2}) & = & C_{R}\left(k_{1},k_{2}\right)-C_{B}\left(k_{1},k_{2}\right)\\ GB(k_{1},k_{2}) & = & C_{G}\left(k_{1},k_{2}\right)-C_{B}\left(k_{1},k_{2}\right)\\ GR(k_{1},k_{2}) & = & -RG(k_{1},k_{2})\\ BR(k_{1},k_{2}) & = & -RB(k_{1},k_{2})\\ BG(k_{1},k_{2}) & = & -GB(k_{1},k_{2}), \end{array}$$

these matrices enable us to speculate the histograms of the entire color differences (R-G, R-B and G-B).

The fuzzy mean process with the normalised inputs is subsequently followed to determine the output ${▵}_{\mathrm{RG}}\left(K_{1},k_{2}\right)$ which equals the one of the three fuzzy mean values closest to the reference value $▵P_{\mathrm{RG}}C\left(k_{1},k_{2}\right)$. These fuzzy variables finally determine the resultant outputs of this filter.

Furthermore, in the recent research conducted by [3], in order to address moderate and highly corrupted grayscale images with Random Valued Impulse Noise, an efficient image restoration technique based on spatially directional adjoining pixels and fuzzy logic is designed. By decomposing a larger image size of impulsive regions into numerous overlapping small patches, the low as well as high density of impulse noise can be estimated. Direction-based fuzzy rules give appropriate reasoning for edge and texture detection in an image. A switching-technique-based fuzzified degree identifies a certain pixel of an image as a noise-free, noisy or edge pixel in the filtering phase.

### 4.3. Fuzzy Vector Partition

To determine the possibility that a pixel is contaminated by impulse noise, fuzzy rule systems with fuzzy inference methods are used. However, it is hard to execute the signal process steps for those subordinate functions to be achieved precisely and for the fuzzy approximate reasoning to be performed accurately. A partition learning method is proposed to deal with such issues [19,50,86]. Assuming a *k*-dimensional observation of vectors O(k) given by *k* one dimensional linguistic variables, a partition is defined so that the observation vector space, *S* sub-set of $R^{K}$, is classified into a set of ς mutual exclusive blocks, defined as $\Omega_{1}$, $\Omega_{2}$,…,$\Omega_{\varsigma }$ [19], satisfying

(18) $$\Omega_{i}=\left\{O\left(k\right)\in S:c\left(O\left(k\right)\right)=i\right\},\;i=1,2,\ldots ,\varsigma ,$$

in which the classifier c(·) is defined as a function of the observational vector O(k). For every input x(k) in correspondence to the O(k), a partition of the vector space *S*, is merely categorized into one of ς non-overlapping blocks as per the c(·). Referring to [19], the ς blocks $\Omega_{i},i=1,2,\ldots ,\varsigma$ satisfy

(19) $$S=\overset{\varsigma }{\underset{i=1}{⋃}}\Omega_{i}\;\mathrm{and}\;\Omega_{i}\cap \Omega_{j}=\emptyset ,\;i\neq j.$$

Owing to its computational efficiency, the scalar quantisation (SQ) is normally considered to be the preferred classifier c(·) when designing the partition fuzzy filter. Every block *i* can be considered as a Cartesian product of the interval blocks $s_{K}$ namely, $i=s_{1}\times s_{2}\times \ldots \times s_{K}$. Then, every scalar component $O_{d}\left(c\right)$ can be categorized in an independent way via SQ [87].

As per the partition of the measurement vector space, the subordinate function for the image element x(k) is written as $\alpha_{i}\left(k\right)$, i=1,2,…,ς. A learning approach is applied to obtain the optimum filter, with the output value as close to the initial signal as possible.

The work in [19,50] proposes a vector partition filter [19,88] to tackle the nonstationary statistics of image structures, and realize the integration of standard deviation with the fuzzy ranking technology [89,90] to minimise the influence of noise-type-related misclassification.

The fuzzy-ordered specimen vector, ${\tilde{S}}_{r}={\left[x_{\left(1\right)}\ldots ,{\tilde{x}}_{\left(N\right)}\right]}^{T}$, is produced as per the centroid de-fuzzification approach through a normalised matrix transformation [89,90], i.e.,

(20) $${\tilde{S}}_{r}=\tilde{R}S_{x}/\left|\right|\tilde{R}\left|\right|$$

where $\tilde{R}$ is produced from the matrix R via the substitution of a real-valued constituent for its binary counterpart written as

(21) $${\tilde{R}}_{\left(i\right)},j=\mu \left[x_{\left(i\right)},x_{j}\right]$$

According to Equation (20), the computation of the fuzzy ordered specimen ${\tilde{x}}_{\left(i\right)}$ is stated below

(22) $${\tilde{x}}_{\left(i\right)}=\frac{\sum_{j=1}^{N}\mu \left[x_{\left(i\right)},x_{j}\right]x_{j}}{\sum_{j=1}^{N}\mu \left[x_{\left(i\right)},x_{j}\right]}$$

in which μ[·]ϵ[0,1] is a fuzzy subordinate function that describe the level to which $x_{\left(i\right)}$ and $x_{j}$ are associated. The subordinate function μ is normally modeled as a Gaussian function based on the specimen spread [89,90].

Now one can use the input variables to calculate the partition cells. When W(c) is a filtering window centered at c and involving *N* image elements, and the multivariable satifies the equation: $x\left(c\right)={\left[x^{R}\left(c\right),x^{G}\left(c\right),x^{B}\left(c\right)\right]}^{T}$, which represents the center image element of the window, and referential filter is completed via a central weighted vector filter with special step-wise preservation attributes [91,92]. The filter is afterwards applied to W(c) for several times to produce the estimates below:

(23) $$Y\left(c\right)={\left[y_{1}\left(c\right),y_{2}\left(c\right),\ldots ,y_{k}\left(c\right),\ldots ,y_{\frac{N-1}{2}}\left(c\right)\right]}^{T}$$

in which $y_{k}\left(c\right)$ reflects the output produced via the referential filter with a central weight $1\leq k\leq \frac{N-1}{2}$.

For a given center weight *k*, the output of the fuzzy ranked CWVMF is written as

(24) $${\tilde{Y}}_{k}\left(c\right)=\frac{\sum_{i=1}^{N}\left\{\mu \left[y_{k}\left(c\right),x_{i}\left(c\right)\right]\cdot x_{i}\left(c\right)\right\}}{\sum_{i=1}^{N}\{\mu \left[y_{k}\left(c\right),x_{i}\left(c\right)\right]}$$

in which $y_{k}\left(c\right)$ is in correspondence to the output of the CWVMF, and $x_{i}\left(c\right)$ is identical to a chromatic specimen of W(c). Thereafter, the formulation of a distance vector is realized at every coordinate *c* as per the fuzzy reference estimates, ${\tilde{y}}_{k}\left(c\right)$, and x(c) form the central image element of the filtering window, according to [91,92].

According to [50], an enhanced form of the partition filter that leverages partition-based trimmed vector median, instead of center-weighted vector median, as a fuzzy reference estimator can be designed and evaluated.

The partition-based trimmed vector median differs from the trimmed vector median that is center-weighted as illustrated in Section 3.2, the difference lies in the fact that the latter replaces the threshold value of center-weighted trimmed vector median with (N−1)/2. The updated version of the fuzzy partition filter simplifies computation and adapts well to local features of image structures. A more recent study performed by Saddam et al. [93] illustrates the effect of fuzzy partitioning in Crohnąŕs disease classification via the use of a neuro-fuzzy based approach. The proposed system with eight partitions shows an accuracy of 97.67% with sensitivity, specificity, positive predictive and negative predictive value of 96.07%, 100%, 100% and 94.61%, respectively. Therefore, such a partition-based fuzzy model can be thought of as a process of dimension reduction in the case of classification, as 95.33% reduction dimension is obtained.

## 5. Fuzzy Filters Design and Implication

Basic concepts on noise detection methods along with basic fuzzy sets for noise filtering were described in Section 3 and Section 4.1. In this section, several recently developed de-noising and classification methods along with the outputs of fuzzy filter systems are discussed. Normally, as a noisy color pixel or component, a filtering or smoothing operation should be carried out proportionally to achieve accurate image analysis. To better estimate the original value while retaining edge information and avoiding color artifacts, the estimated value is calculated utilizing information from its local neighborhood or the other color components of the filtered pixels.

### 5.1. Fuzzy Cellular Automata for Noise Removal

One of the non-linear filtering techniques, cellular automata, has been utilized to eliminate image noise using fuzzy logic. Space is defined in cellular automata as a uniform grid containing several components known as cells. For computer science applications such as image processing, cellular automata can be utilized to model a wide range of phenomena. In cellular automata, the value of each cell is decided exclusively by its adjacent surrounding cells, with discrete time as well as local and consistent rules. Using a sliding window, cells can take a new value at different time steps (t = 0, 1, 2, …, n) according to their current positions and their neighbors by following a local transition function. Cellular automata are defined by the four tuples L, Q, r, f, where “Q” is a finite set of states, “r” and “f” are neighborhood radius and a transition function, respectively. The letter “L” denotes the regular grid of cells.

**Figure 2:**
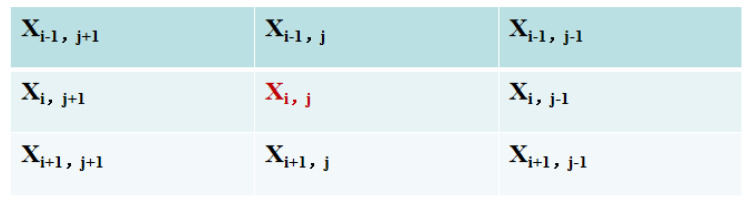
Mean cell location in Moore neighborhood architecture [94].

The adjacent structure of two-dimensional cellular automata, as shown in Figure 2, depicts the core cell $x_{i,j}$ and its eight surrounding neighbors. Depending on the transfer function F and the cell state of its eight surrounding neighbors at time *t*, the cell status at time t+1 is modified.

Piroozmandan et al. [94] presented a fuzzy-logic-based local transmission function to achieve fuzzy cellular automata, with the intention of eliminating image impulse noise while maintaining essential details in the image, such as the edges and texture. Fuzzy cellular automata (FCA) was described as a strong tool in artificial intelligence through [95]. For cell states and functional traction areas, fuzzy values in form of linguistic variables are employed instead of definite values in this framework.

**Figure 3:**
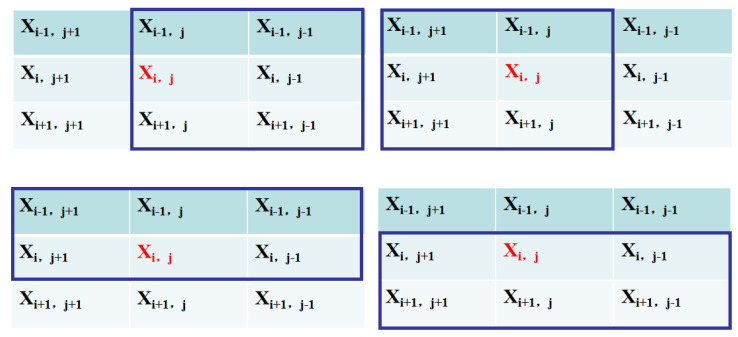
Illustration of four potential placements of the five adjacent pixels around the red key pixel [94].

To achieve fuzzy cellular automata, a fuzzy logic-based local transmission function aims at the removal of impulse noise in an image and maintaining the meaningful details such as the edges and texture of the image [94]. A two-stage method is presented for monitoring impulsive noise and image restoration. In the first stage, damaged pixels are located in two steps. The initial approach is to identify the damaged pixels by estimating the least value of its core pixels and mean value of Moore neighboring pixels. As shown on Figure 3, they are the images computed in terms of the harmonic mean (HM) [96] of the four multiple positions of the five bordering pixels around the key (red) pixel.

The Moore neighbor algorithm is used incorporated with the mean filter for noise detection and feature extraction. Furthermore, the Moore Neighbor algorithm is robust to detect the noise of object as well as features of an object.

The second stage requires an inspection of pixels that were detected as uncorrupted in the previous stage, in order to ascertain if they are still uncorrupted or have been damaged. For the uncorrupted pixel detected, one measures the extreme values of the arithmetic mean (AM) of four separate places of five pixels in the vicinity of the key point on the basis of Figure 3. The idea is simple but effective for noise detection. Noise basically occurs where there shows significant changes in intensity. The principle of the algorithm used is based on detecting an increase in the difference between those pixels where intensity values change significantly.

Finally, the damaged and corrected pixel is diagnosed using cellular automata as follows [94]:

(25) $$\begin{array}{c} X_{j,j}=\left\{\begin{array}{c} X_{j,j}\mathrm{is}\;a\;\mathrm{uncorrupted}\;\mathrm{pixel},\;F\left(i,j\right)=x_{HM/AM}^{max}<=x_{i,j}\\ N-1,N-2\;\mathrm{is}\;a\;\mathrm{corrupted}\;\mathrm{pixel},\;F\left(i,j\right)=x_{HM/AM}^{min}>x_{i,j} \end{array}\right. \end{array}$$

**Figure 4:**
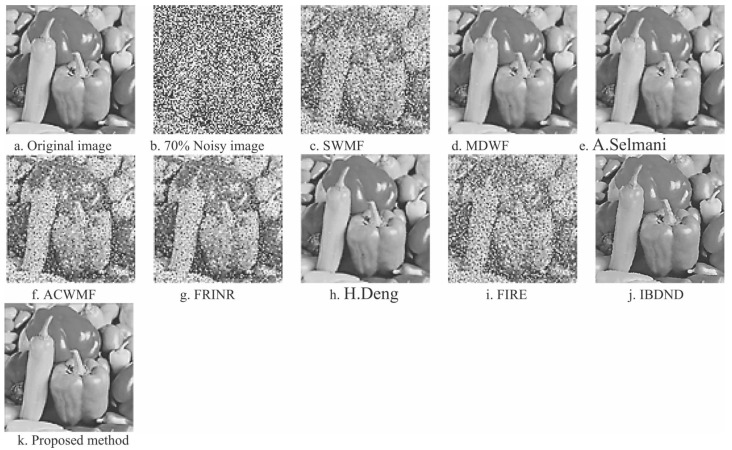
Illustration of multiple filtering strategies for the Peppers image with 75% salt-and-pepper noise [94].

**Figure 5:**
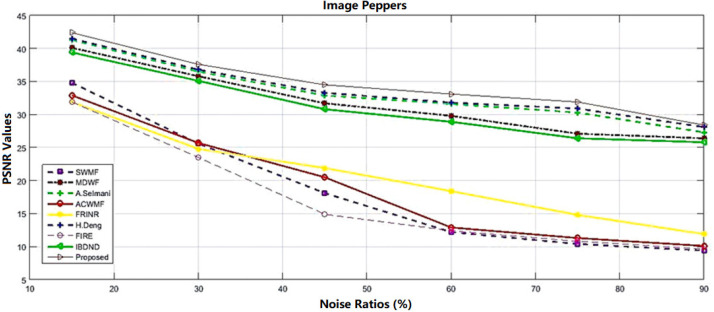
PSNR measurements on Peppers reconstruction image with 768×768 pixels by 15–90% salt-and-pepper noise [94].

Figure 4 and Figure 5 respectively demonstrate the outcome of the filtered Peppers image consisting of 768×768 pixels with a 75% salt-and-pepper noise density, along with the PSNR graph for the Fuzzy based solution compared to some other strategies, for example, adaptive impulse sensing via center-weighted median filters (ACWMF) [97], new impulse detector regarding switching median filtering (SWMF) [98], Modified directional weighted filter (MDWF) [99], A. Selmani’s method [100], Fuzzy stochastic stimulus-related noise removal method (FRINR) [28], H. Deng’s method [101], fuzzy inference rule by the else-action filter (FIRE) [36] and boundary discriminative noise detection (BDND) [102], along with a two-phase fuzzy cellular automata approach proposed by [94].

From Figure 4, the subjective quantitative measurement shows great visual performance with clear edges in the Peppers image restoration using the proposed filtering in regard to two-phase fuzzy cellular automata (Figure 4k). The proposed fuzzy approach allows edges to be restored even in the low contrast areas, where fuzzy rule adopted was adjusted to obtain good results, and identify the edges of the image. Even though the methods such as SWMF (Figure 4c), ACWMF (Figure 4f), and FIRE (Figure 4i) remove a lot of salt-and-pepper noise in the images, they are not successful in preserving image details such as edges, especially in the regions with low contrast.

### 5.2. An Optimized Fuzzy System for Edge Detection

When it comes to digital image processing, edge detection is a commonly used approach. The target is to detect pixels that correlate the image edges. The employment of filters to attenuate noise leads to loss in edge detection capability. It is crucial to evaluate the intensity restrictions of pixels in their vicinity in order to promote edge detection. Many spots in an image have an opaque gradient, but none of them constitute of joint edges of space. To determine the edge points, some linear and nonlinear algorhitms, such as Sobel, Prewitt and Robert, have to be adopted. As fuzzy approaches have been gaining more popularity recently, they may soon become one of the most successful methods for edge detection tasks.

Azimirad et al. [103] designed efficient Prewitt mask matrices and proposes an optimum rule-based fuzzy inference system. Table 1 improved fuzzy set theory-based edge detection rules for Prewitt mask matrices. There are four inputs ($PI_{i}$, 1 = 1, 2, 3, 4) and one output in the designed fuzzy system. In this table, “Bl” is black pixel value, “Wh” is white pixel value and “En” is edge enable. This table demonstrates the 16 fuzzy rules offered on the [103].

**Figure 6:**
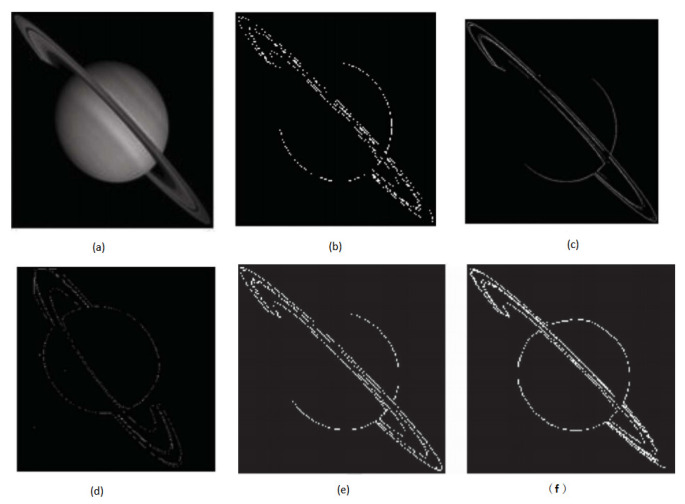
Illustration of the simulation outcomes for classic edge detection techniques: (**a**) Original Image, (**b**) Sobel Filter, (**c**) Prewitt Filter, (**d**) Robert Filter, (**e**) fuzzy approaches designed by Deepak [104], (**f**) Optimised fuzzy system designed by Azimirad et al. [103].

Deepak et al. [104] compared the classic edge detection algorithms, including Sobel Filter, Prewitt Filter, Robert Filter, along with two new fuzzy approaches. Totally 34 fuzzy rules were defined. The proposed fuzzy system was able to detect the edges of the image more reliably, and aid in improving the sharpness and clarity of the edges, according to the results. It has a more accurate and more reliable performance in edge detection with the comparison to the traditional methods. The results of simulations utilizing classic edge detection approaches and fuzzy-based modelling for edge identification are shown in Figure 6. It is obvious to see that fuzzy based filtering performs well in the edge detection tasks as shown in Figure 6e,f, compared with traditional filtering approaches as shown in Figure 6b–d.

Generally, these traditional filters process the data in a relatively short time and are computationally optimized, however, they are susceptible to noise. The Fuzzy method performs mathematical and logical reasoning based on approximations rather than crisp values. Therefore the technique significantly reduces the complexity of problems where fixed values cannot be attained or predicted.

### 5.3. Fuzzy Deep Ensemble Classifier

**Figure 7:**
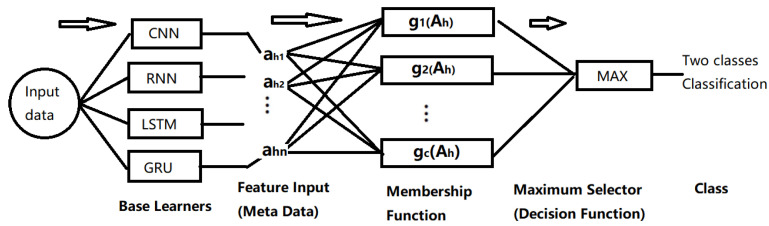
Block diagram of the proposed Ensemble model. After [105].

The main goals in AI based image processing is the creation of an efficient classifier. In most domains, the deep learning approach holds a significant place in contemporary research. The fuzzy logic technique, on the other hand, is directly analogous to the segmentation approach. Das et al. [105] designed a deep ensemble learning based on a fuzzy min-max (FMM) classifier [106]. The outcome merges the structure of four deep learning-based models, namely Convolutional Neural Networks (CNN), Recurrent Neural Network (RNN), Long Short Term Memory (LSTM) based network, and Gated Recurrent Unit (GRU) based neural network, all of which were trained simultaneously from the same dataset, (Ah). The use of a fuzzy min-max classifier to predict the performance of the underlying classifiers reduces the uncertainty in performance. The block diagram of the proposed method is depicted in Figure 7. The input dataset Ah is passed through each of the four proposed classifiers that are deep neural network related, and the four neural network approaches produce different discriminant functions used for feature extraction and object classification. The values of the resultant classification function are compared and the one with the largest value is selected to identify the pattern class.

**Figure 8:**
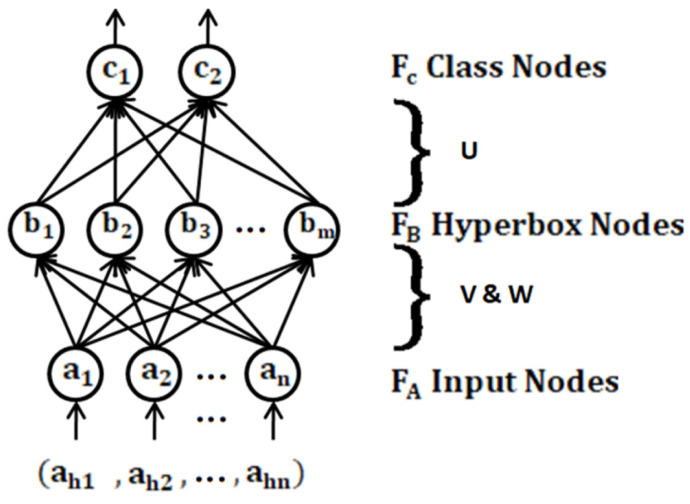
Three-layer fuzzy neural network [105].

The fuzzy min-max (FMM) classifier includes one input layer, one hidden layer, and one output layer. The Fuzzy model receives the eight-element vector coming from the four deep learning classifiers with two classes of each. The second layer FB consists of the hyper box based fuzzy set nodes (b1, b2, *…*, bm). A min-max learning procedure with a fuzzy membership function is used to create dual links between input nodes and hyper box nodes by considering jth hyper box as a fuzzy membership function $B_{j}=V_{j},W_{j},Y_{j}$, where, $V_{j}$, $W_{j}$ and $Y_{j}$ regard the mini point, max point, and the corresponding label of the class, respectively. The final layer FC consists of class nodes (c1, c2). The layer configurations of the FMM model are depicted in Figure 8.

**Figure 9:**
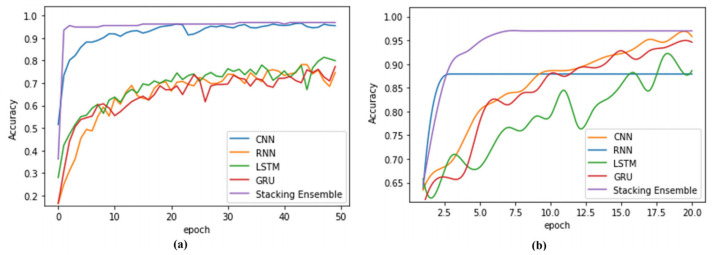
The comparison of test accuracy for (**a**) Kaggle Brain image dataset (**b**) Chest X-ray dataset [105].

The research work by Das et al. [105] also looked at the fidelity of the mentioned four base learning models in association with neural networks along with the stacked ensemble learning model (shown as Figure 9). The ensemble output from the base classifiers is fed to the fuzzy model in terms of class probability and labels. The min-max algorithm for correct decisions is used in the fuzzy model. The stacked ensemble outperforms base models, increasing final accuracy in contrast to base models for both datasets, for example, 97.62% validation accuracy on the brain tumor classification using the Kaggle dataset whereas 95.24% was achieved on the chest X-ray dataset. When evaluated on the benchmark BRATS dataset, the Fuzzy deep ensemble model likewise delivers competitive results with an accuracy of 97.59%.

### 5.4. Fuzzy Non Local Mean Filter

Non local Means (NLM) filters [107] with bias correction is a promising technique for signal dependent noise. The NLM denoising is based on self-similarity and is computationally very expensive. A method called lifting was recently demonstrated to conduct NLM denoising of one-dimensional signals [108]. Owing to the independent effect of the patch length, the cost of lifting is dramatically reduced, especially for large patches. Unfortunately, it is difficult to directly extend lifting for non-local means denoising of images.

Singha and Kaurb [107] designed a fuzzy-rule based fast non-local mean filtering, and is based on a speed enhanced NLM. The fuzzy Jacord similarity measurement on integral image is in terms of the self-similarity of sub windows. This helps to find the weights of similar pixel at a faster rate than the traditional NLM algorithm and more accurately than the existing fast NLM method. These similar pixels further generate noise-free pixels using conventional bias subtraction methods. The fuzzy non local mean filter performs better than existing Fast NLM technique with high density Rician noise in standard brain MR images and is 20 times faster than traditional NLM.

The other fuzzy filters, such as fuzzy-bilateral filtering [109,110], enables an interlaced field to progressive frames to be converted while sustaining and improving image details, the key operations of image processing. However, it is a challenge to perform filter-based interpolation and detail enhancement simultaneously, as they are contradictory operations. Since the fuzzy-bilateral filter [109] prefers close pixels over distant pixels in terms of both domain and range, it allows adaptive application onto both existing pixel activity and the associated position between existing neighbor pixels and the missing pixels. Simulation results on video analysis and reconstruction prove the possibility of achieving efficient interpolation of the interlaced field while enhancing details.

### 5.5. Resultant Experiments and Comparison

**Figure 10:**
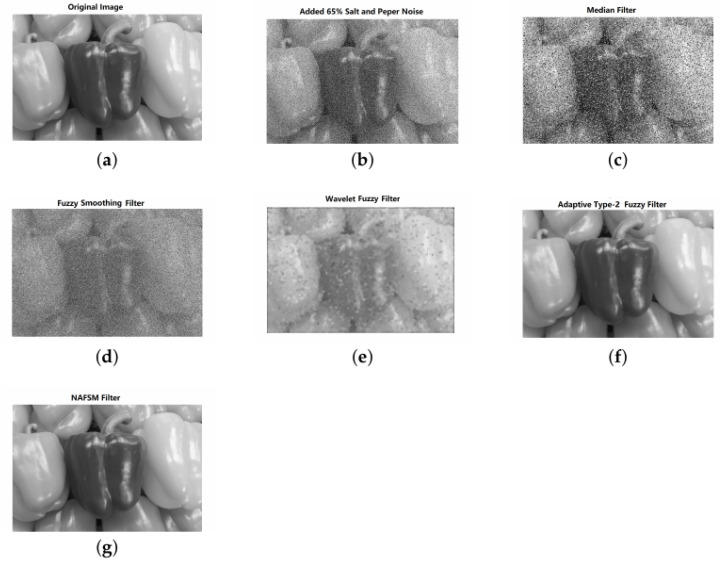
Illustration of multiple filtering strategies for Peppers image with 65% salt-and-pepper noise. (**a**) An original image with image size of 800×1200. (**b**) A noisy image with added 65% salt-and-pepper noise. (**c**) Filtered image using a median filter. (**d**) Filtered image using a fuzzy smoothing filter. (**e**) Filtered image using a fuzzy wavelet filter. (**f**) Filtered image using adaptive fuzzy type 2 filter. (**g**) Filtered image using NAFSM.

The test image, i.e., Peppers with image size of 800×1200 shown in Figure 10a has been used to evaluate the performance among several filtered images with added 65% salt-and-pepper noise as shown in Figure 10b. These filtered images include an median filtered image, a fuzzy smoothing filter, a fuzzy wavelet filter, adaptive fuzzy type 2 filter [111], and a noise adaptive fuzzy switching median filtered image (NAFSM) [112]. The median filter shows difficulty in removal of salt-and-pepper noise, fuzzy smoothing enables to partly remove the noise, but at the cost of image blurring, which in turn loses sharp edges. Wavelet fuzzy filter is capable of protecting the image boundary, but at the loss of some small parts of an image. Adaptive fuzzy filter and NAFSM filter have the ability to effectively filter the noise in an image, with the noise to be filtered very thoroughly.

The filter performance is assessed by taking into account both the noise suppression and the detail preserving capabilities of the filter. To this end, we have used the Mean Square Error (MSE), the Peak Signal to Noise Ratio (PSNR), and the Signal to Noise Ratio (SNR) that measure the detail preserving capability, the noise suppression capability, and the strength of a desired signal relative to background noise, respectively.

In Table 2, the NAFSM filter reflects its superior performance in terms of MSE, PSNR, SNR, this is closely comparable to the performance achieved in impulse noise detection and reduction approaches. Adaptive fuzzy type 2 filter (AFT2F) shows slightly weakened performance in image restoration compared with NAFSM filter, but with improved results compared with fuzzy smoothing and fuzzy wavelet filtering. Fuzzy smoothing shows an inferior performance, in impulse noise removal tasks.

Table 3 is to compare the performance in terms of the state of art results using conventional filters, fuzzy filters, along with deep learning filters. The images include color images, gene datasets, satellites images, and MRI datasets. Noise types include salt-and-pepper noise (NM1), Impulse noise (NM2), Gaussian & Impulse noise (NM3), and Rician noise decreasing the MR image quality.

## 6. Conclusions and Future Work

This paper has reviewed some recent advances in the analysis and synthesis of fuzzy-model-based nonlinear filters. Different noise models for the evaluation of the fuzzy filter performance were considered. Furthermore, different fuzzy rule designs based on local information are also discussed. The construction of an optimal fuzzy system for edge detection has received special attention, such as fuzzy cellular automata for the identification of corrupted pixels towards image noise removal, a fuzzy deep ensemble classifier with the biomedical application. Various results on the image visualised analysis of fuzzy-model-based filter design for edge detection, denoise removal and deep ensemble classifier, have been highlighted during the survey. Based on the literature review, some related issues for future research works are outlined as follows.

As a pre-processing step for compaction, edge recognition, image segmentation, the removal of random-valued noise (RVN) and salt-and-pepper noise (SPN) from digital images are critical [4,116,117]. Fuzzy-model-based nonlinear systems have been developed to reduce combined noise from digital images. The existing results on analysis and design of fuzzy-model-based nonlinear systems were derived mainly in terms of sufficient conditions with the use of various mathematic models, such as neural networks, cellular automata, fuzzy logic controllers, and so on. How to further reduce the conservatism and relax the computation complexity, as well as maintain the important image details deserve further investigation.

In the past few years, sophisticated technologies such as fuzzy-rule based systems, neural networks and numerous optimization methods have been utilized to detect edges and reduce noise in digital images. These techniques are effective at detecting noise in high-noise environments, but they have several drawbacks. When the density of digital photos is large, these methods introduce computational complexity, and key image features may be lost, as well as certain image edges. Although there have been some attempts to deal with those issues, the obtained results are still preliminary as most of the useful information on membership functions is ignored during the networked fuzzy controller/filter design. Thus, some more powerful relaxation techniques and compensation strategies to tackle this issue are desired.

Researchers working on a variety of image detection problems are still looking for an efficient classifier. In recent advancements, it has been noticed that most works are primarily focused on CNN-based models, whilst alternative deep learning strategies such as the fuzzy-model-based nonlinear classification scheme are limited, despite the fact that this technique outperforms a single model. Fuzzy-model-based nonlinear classification systems with reduced fault classification and reduced fault-tolerant control problems for various image processing, especially as applied to medical image processing tasks would be an interesting topic [118,119,120,121].

Nowadays, big data are present in almost everywhere of our daily life including social networks, online and offline transactions, medical records, and sensors. An immense volume of heterogeneous data can be generated at exponential rates. The capabilities of handling big data are vital to many scientific and engineering applications. Fuzzy set techniques play an important role in processing big data as they can not only model uncertainties of both data sources and results of algorithms, but offer a platform for potentially new application areas where fuzzy-rule based logic control has not yet been deployed. Examples are likely to be found in emergent research areas such as virtual and augmented reality. Furthermore, it may also be argued that they may also be incorporated to some degree in many future solutions for business, education, robotics, AI based diagnostics or manufacturing, and renewable energy sectors, as long as DSP based filter engineering approaches may be seamlessly integrated within these fields.

## Figures and Tables

**Table 1:** Displays the new and improved fuzzy set theory-based edge detection rules.

Input				Output
PI1	PI2	PI3	PI4	Out
Wh	Bl	Bl	Bl	En
Bl	Wh	Bl	Bl	En
Bl	Bl	Wh	Bl	En
Bl	Bl	Bl	Wh	En
Bl	Bl	Bl	Bl	En
Bl	Wh	Wh	Wh	En
Wh	Bl	Wh	Wh	En
Wh	Wh	Bl	Wh	En
Wh	Wh	Wh	Bl	En
Wh	Wh	Wh	Wh	Wh
Bl	Bl	Wh	Wh	En
Bl	Wh	Bl	Wh	En
Bl	Wh	Wh	Bl	En
Wh	Bl	Wh	Bl	En
Wh	Wh	Bl	Bl	En
Wh	Bl	Bl	Wh	En

**Table 2:** Comparison of the performance measured in terms of MSE, PSNR and SNR using the Peppers image contaminated with 65% noise.

Fuzzy Filters	Fuzzy Smoothing	Wavelet Fuzzy Filter	AFT2F	NAFSM
MSE	189.35	107.18	5.9392	2.3532
PSNR	15.3587	17.8798	40.3935	44.4141
SNR	10.7782	12.9507	16.3254	20.3461

**Table 3:** Comparison of the performance in terms of the state of art results using conventional filters, fuzzy filters, along with deep learning filters.

Filter	Image	NT	ND	MAE	MSE	NCD	PSNR	SSIM	FSIM	Sens	Spec	Accu
VMF	Lena	NM2	10%	3.69	56.5	4.29						
BVDFs	Lena	NM2	10%	4.10	67.6	4.32						
DDFs	Lena	NM2	10%	3.73	57.3	4.24						
AVDF	Lena	NM2	10%	4.54	59.5	5.0	3					
HMAMF	Lena	NM2	10%	5.67	91.98							
HVMF	Lena	NM2	10%	3.59	39.16							
CWTVM	Lena	NM2	10%		21.80							
CWVM	Lena	NM2	10%		25.9							
SCWVMF [113]	Boats	NM2	10%	3.89	112.54		27.62					
PRF [53]	Peppers	NM4	10%	8.54		8.44	25.44					
AVMF	Peppers	NM4	10%	7.93	8.04	26.59						
PGSVMF	Peppers	NM4	10%	8.17	9.01	26.36						
PGSAMF	Peppers	NM4	10%	8.36	8.25	27.28						
FMPGSAMF	Peppers	NM4	10%	8.33	8.48	26.23						
FRVP	Parrot	NM2	10%	0.90	24.4	0.57						
ACWVDF	Parrot	NM2	10%	0.72	37.4	0.37						
DPGF [56]	Lena	NM2	20%				32.2					
DWM [62]	Lena	NM2	20%				33.6					
AVDDF [44]	Lena	NM4	FPGA				1.00	33.34				
CA+QFT [42]	SI	SIN			25.24			2.77	0.38			
FWMF [82]	Lena	NM2	20%				39.5	0.98	0.997			
FINR [83]	Parrot	NM2	20%				2.37	32.92				
AFSF [114]	Parrot	NM2	20%				2.86	30.53				
HAF	Parrot	NM2	20%				9.28	25.84				
PGSF [54]	Parrot	NM2	20%				4.18	28.48				
FISF	Parrot	NM2	20%				3.01	29.23				
QSAF	Parrot	NM2	40%				35.33	0.939	0.972			
FIDRM [115]	Parrot	NM2	40%				35.13	0.88	0.97			
FCA [94]	Lena	NM2	15%				34.7	0.98				
SWF	Pepper	NM1	30%				18.1	0.79				
ACWMF	Pepper	NM1	30%				20.5	0.87				
FCA	Pepper	NM1	30%				42.5	0.999				
FANLM [107]	BMRI	Rician	9%				30.5	81.5				
NLM [107]	BMRI	Rician	9%				28.1	73.2				
AT2F [111]	Lena	NM2	20%				40.79					
FARTMAP [105]	BH									0.99	0.98	98.7
RNN	BH									0.90	0.79	87.2
LSTM	BH									0.92	0.86	90.38
GRU	BH									0.92	0.83	89.7
NFP [93]	GD									96.07	100	97.67

## Data Availability

Not applicable.

## Abbreviations

The following symbols and abbreviations are used in the manuscript:

I	a two-dimensional (2-D) image
$\left[x_{\left(k_{1},k_{2}\right)}\right]$	a two-dimensional (2-D) image matrix
*H*	the number of rows of an image matrix
*W*	the number of columns of an image matrix
$x_{\left(k1,k2\right)}$	a pixel addressed by 2-D sequence
$x_{k}$	a pixel addressed by 1-D sequence
*L*	individual channel of a color image
R,G,B	red, green, blue color components
$\upsilon_{k}^{L}$	model the original pixel
ι	contamination component
*p*	the sample corruption probability
W	a support window
*N*	commonly used filter window size
*c*	center pixel position under a window
*l*	gray levels
$y_{k}$	the output color vector
γ	norm parameter
$L_{1}$	norm of Block distance
$L_{2}$	norm of Euclidean distance
$L_{\infty }$	norm of Max distance
$d_{i}$	an aggregated distance of a specific pixel
$d_{\mathrm{VMF}}$	an aggregated distance of VMF
$d_{\mathrm{VDF}}$	an aggregated distance of VDF
$d_{\mathrm{DDF}}$	an aggregated distance of DDF
$d_{\mathrm{PG}}$	accumulated similarity of peer group
ρ	distance function
$y_{VM}$	output of vector median filters
$y_{VD}$	output of vector directional filters
$y_{BVD}$	output of basic vector directional filter
$y_{\mathrm{HM}}$	output of hybrid multichannel filter
$y_{\xi }$	output of identification filter
$y_{L_{\mathrm{mod}}}$	modified L filter output
$y_{\mathrm{FPG}}$	output of fuzzy *peer group*
α, β	coefficients of hybrid multichannel filter
$R_{i}^{w}$	center weighted aggregated vector distance
*w*	a positive integer central weight
$y_{w}\left(c\right)$	output of a CWVM filter
$e_{w}\left(c\right)$	center pixel reference distance
*ℏ*	a group of preset thresholds
T	threshold value
$\hat{y}$	switch-based center weight vector median
P(x(c),r,d)	peer group
*T*	triangular norm
*S*	triangular conorm
μ	membership function
C	certainty
$\Psi_{i}$	number *i* partition cell
Ψ	fuzzy matrix
σ	sample spread
Ξ	Laplacian mask
$\nabla_{D}$	basic gradient value
${\dot{\nabla }}_{D}$	related gradient values
${\ddot{\nabla }}_{D}$	related gradient values
*D*	gradient direction within a sliding window
SVM	support vector machine
DDMM	Distinct distance measure metrics
BVDF	Basic vector directional filter
GVDF	Generalised vector directional filter
DDF	A directional distance filter
FPGA	Field programmable gate array
AVDDF	Adaptive vector directional distance filter
HMF	Hybrid multichannel filter
CWVMFs	Center weighted vector median filters
PRT	Peer group filter
FPGF	Fast peer group filter
ACWMF	Adaptive center weighted median filters
SWMF	Switching median filtering
MDWF	Modified directional weighted filter
FRINR	Fuzzy stochastic stimulus related noise removal method
FIRE	Fuzzy inference rule by the else-action filter
BDND	Boundary discriminative noise detection
FMM	Fuzzy min max
CNN	Convolutional Neural Networks
RNN	Recurrent Neural Network
LSTM	Long Short Term Memory neural network
GRU	Gated Recurrent Unit
NLM	Non local Means
NAFSM	Noise adaptive fuzzy switching median filtered image
MSE	Mean Square Error
PSNR	Peak Signal to Noise Ratio
SNR	Signal to Noise Ratio
AFT2F	Adaptive fuzzy type 2 filter
VDF	Vector directional filter
SSIM	Structured Similarity Index
QFT	Quaternion Fourier Transform
DDF	Directional-distance filters
NCD	Normalized color difference
HMF	Hybrid multichannel filter
HMAMF	Hybrid marginal arithmetic mean filter
HVMF	Hybrid Vector mean Filters
AVMF	Adaptive vector median filter
PGSVMF	Peer group switching vector median filter
PRF	Peer region filter
PGSAMF	Peer group switching arithmetic mean filter
FMPGSAMF	Fuzzy modified peer group switching arithmetic mean filter
WVDFs	Weighted vector directional filters
HVFs	Vector directional hybrid filters
DPGF	Directional weighted peer group
FWMF	Fuzzy based Weighted Mean Filter
FSIM	Feature similarity index
MWMF	Modified Weighted Mean Filter
FINR	Fuzzy based impulse noise reduction method
PGSF	Peer group switching filter
FISF	Fuzzy inference system filter
HAF	Histogram adaptive fuzzy filter
FIDRM	Fuzzy impulse noise detection and reduction method
QSAF	Quadrant based spatially adaptive fuzzy filter
FRVP	Fuzzy-rank vector partition
FCA	Fuzzy cellular automata
ADCWM	Adaptive impulse detection via center weighted median filters
SWF	Switching median filter
FARTMAP	Fuzzy adaptive resonance theory mapping
FANLM	Fuzzy Cmeans and adaptive non-local means
UNLM	Unbiased NLM filter
AT2F	Adaptive Type-2 Fuzzy Approach
